# An industrial engineering approach to laboratory automation for high throughput screening

**DOI:** 10.1155/S1463924600000225

**Published:** 2000

**Authors:** Karl C. Menke

**Affiliations:** Sphinx Pharmaceuticals A Division of Eli Lilly and Company Research Triangle Park NC USA

## Abstract

Across the pharmaceutical industry, there are a variety of approaches to laboratory automation for high throughput screening. At Sphinx Pharmaceuticals, the principles of industrial engineering have been applied to systematically identify and develop those automated solutions that provide the greatest value to the scientists engaged in lead generation.


Journal of Automated Methods & Management in Chemistry, Vol. 22, No. 5 (September–October 2000) pp. 143–144

### An industrial engineering approach to laboratory automation for high throughput screening

**Karl C. Menke**

*Sphinx Pharmaceuticals, A Division of Eli Lilly and Company, Research Triangle Park, NC, USA*

*Across the pharmaceutical industry, there are a variety of approaches to laboratory automation for high throughput screening. At Sphinx Pharmaceuticals, the principles of industrial engineering have been applied to systematically identify and develop those automated solutions that provide the greatest value to the scientists engaged in lead generation.*

*The Handbook of Industrial Engineering* defines industrial engineering as the field 'concerned with the design, improvement and installation of integrated systems of people, materials, equipment and energy.' This description is certainly applicable to the discipline that has become known as High Throughput Screening (HTS). Many approaches to laboratory automation for HTS have focused on a single aspect, be it robotics, miniaturization, or a particular detection format. A much more successful approach recognizes the necessary integration of not only hardware and software, but of people, materials and processes as well.

At Sphinx Pharmaceuticals, this approach has been organized using operation analysis and targeted process improvements. Operation analysis has involved stratifying assays according to their complexity, throughput, assay type and unit operation (figure 1).

With the proliferation of new detection technologies now available, the number of potential assay permutations continues to increase. When one considers the variables of biochemical vs. cell based, number of reagent additions, type and number of incubations, plate format and signal detection method, the number of possible assay configurations exceeds 10 000. However, as shown in figure 2, as few as twelve different unit operations can describe every assay run at Sphinx over the past two years.

Once unit operations have been identified, the suitability of the equipment available to perform those operations can be assessed. Conducting an equipment standardization exercise has the following benefits.

- Identify standard equipment for future purchases.
- Identify equipment types that need investigation/development.
- Prioritize integration and development of tools and methods.
- Reduce complexity and proliferation of required support skills.
- Consolidate sourcing and service.
- Common equipment tool box for screen development.
- Process for evaluating new equipment.

If the workstation approach to automation is utilized, then unit operations can be decoupled. Capacity can be assessed independently for each unit operation. Process improvements can be targeted at specific unit operations, with minimal or no impact on other unit operations.

### Summary

*Automate the process, not the assay*

Of course, this requires knowledge of the process. Assay specific improvements will have short term impact. Process improvements will continue to provide benefits as long as that portion of the process continues.

*Identifying unit operations is the key*

Once assay processes are broken down into unit operations, each unit operation can be standardized and optimized. Capacity can be assessed by unit operations and assays can be assembled.

*Standardize until it hurts . . . but be flexible*

The attitude should be 'Why can't we standardize this method?' There will always be assays that require exceptions, but those exceptions should be justified, and undertaken not just for the sake of doing things differently. In fact, standardizing wherever possible will make it easier to be flexible on those issues that really matter.

- **Simple** – AIR (Add, Incubate, Read) assays; 60,000 compounds/day
- **Moderate** – Radiation handling, plate sealing; 30,000 compounds/day
- **Complex** – Plate washing, reaction transfers, filtration; 15,000 compounds/day

*Figure 1. Stratification of assays by complexity.*

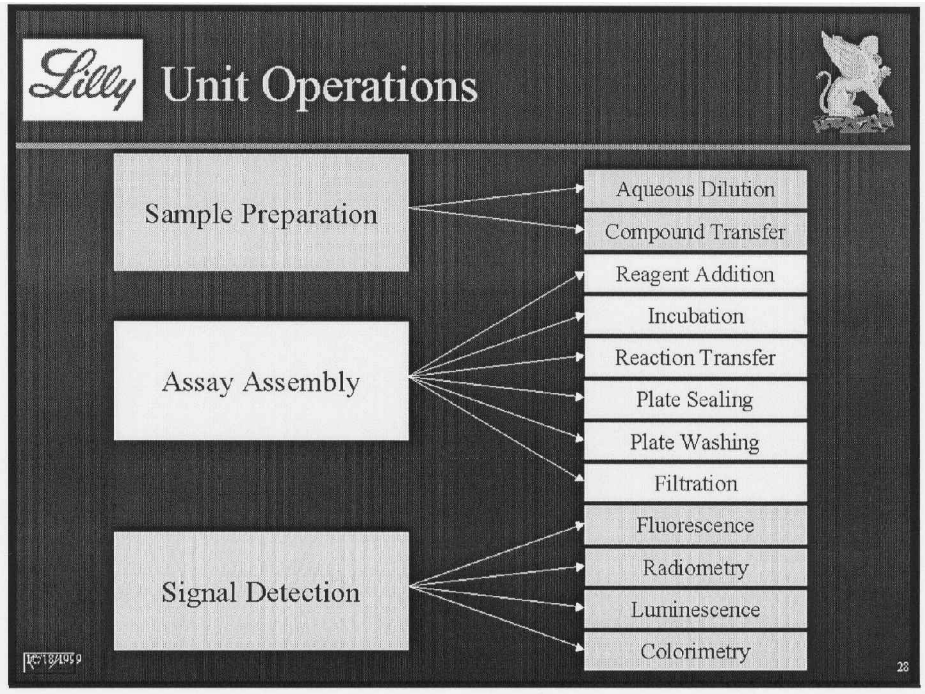

*Figure 2. Assay unit operations sorted into three groups: sample preparation steps, assay assembly steps and signal detection.*

*Look for the bottlenecks*

Using the concepts of cycle time and throughput will allow the determination of the bottlenecks in the process. Targeting process improvements at these bottlenecks will provide true increases in capacity and capability.

*Remember the bottom line*

Ideally, laboratory automation will provide more consistent and reproducible results than manual operations. However, the principal driver behind automation will always be cost. If the *total* life cycle cost of an automation project does not provide a cost advantage, it should probably not be pursued.

### Acknowledgements

The author thanks the members of the HTS Automation Group at Sphinx Pharmaceuticals.

